# Nuclear factor (erythroid derived 2)-like 2 activation increases exercise endurance capacity via redox modulation in skeletal muscles

**DOI:** 10.1038/s41598-017-12926-y

**Published:** 2017-10-10

**Authors:** Sechang Oh, Shoichi Komine, Eiji Warabi, Kentaro Akiyama, Akiko Ishii, Kazunori Ishige, Yuji Mizokami, Keisuke Kuga, Masaki Horie, Yoshihiro Miwa, Takao Iwawaki, Masayuki Yamamoto, Junichi Shoda

**Affiliations:** 1 The Center of Sports Medicine and Health Sciences, Tsukuba University Hospital, Tsukuba, Ibaraki 305-8576 Japan; 2 Faculty of Medicine, University of Tsukuba, Tsukuba, Ibaraki 305-8575 Japan; 3 Department of Life Science, Medical Research Institute, Kanazawa Medical University, Uchinada, Kahoku, Ishikawa 920-0293 Japan; 4 Department of Medical Biochemistry, Tohoku University Graduate School of Medicine, Sendai, Miyagi 980-8575 Japan; 5 Japan Society for the Promotion of Science, Tokyo, 102-0083 Japan

## Abstract

Sulforaphane (SFN) plays an important role in preventing oxidative stress by activating the nuclear factor (erythroid derived 2)-like 2 (Nrf2) signalling pathway. SFN may improve exercise endurance capacity by counteracting oxidative stress-induced damage during exercise. We assessed running ability based on an exhaustive treadmill test (progressive-continuous all-out) and examined the expression of markers for oxidative stress and muscle damage. Twelve- to 13-week-old Male wild-type mice (*Nrf2* ^+/+^) and Nrf2-null mice (*Nrf2* ^−/−^) on C57BL/6J background were intraperitoneally injected with SFN or vehicle prior to the test. The running distance of SFN-injected *Nrf2* ^+/+^ mice was significantly greater compared with that of uninjected mice. Enhanced running capacity was accompanied by upregulation of Nrf2 signalling and downstream genes. Marker of oxidative stress in SFN-injected *Nrf2* ^+/+^ mice were lower than those in uninjected mice following the test. SFN produced greater protection against muscle damage during exhaustive exercise conditions in *Nrf2* ^+/+^ mice than in *Nrf2* ^−/−^ mice. SFN-induced Nrf2 upregulation, and its antioxidative effects, might play critical roles in attenuating muscle fatigue via reduction of oxidative stress caused by exhaustive exercise. This in turn leads to enhanced exercise endurance capacity. These results provide new insights into SFN-induced upregulation of Nrf2 and its role in improving exercise performance.

## Introduction

The production of reactive oxygen species (ROS) increases during muscle contraction^1,2^. Although biological systems are able to detoxify ROS and their reactive intermediates, sustained elevation in ROS levels during repetitive or prolonged muscle contractions may produce imbalances in normal redox states, generating oxidative stress^3^. Exhaustive exercise training in particular promotes bursts in ROS production^4^. Prolonged exercise causes structural damage and contractile dysfunction in skeletal muscle^3,5^. In this manner, redox disturbance within skeletal muscles leads to progressive muscular weakness and fatigue (*i*.*e*. reduced force capacity and slowed contraction rate). These phenomena manifest as reduced exercise endurance capacity^2,3,5–7^.

Recent studies highlight the role of transcription factor nuclear factor (erythroid derived 2)-like 2 (Nrf2) in expression of antioxidant response element (ARE)-driven endogenous antioxidant/xenobiotic detoxifying enzymes, and the role of Nrf2 in attenuating oxidative damage^8^. Modulation of Nrf2-related antioxidant signalling may preserve redox and functional homeostasis in tissues and organs^9^.

Sulforaphane (SFN), a chemopreventive compound produced by the Brassicaceae family, is a potent activator of Nrf2^10^. Many studies suggest that SFN-induced Nrf2 activation may exert cytoprotective effects on various organs damaged by oxidative stress^11^. Malaguti *et al*.^12^ reported that SFN modulates the redox environment in muscle and attenuates damage to skeletal muscle via the Nrf2-ARE pathway. The antioxidant activity of SFN may contribute to increased exercise endurance capacity by reducing muscle fatigue caused by exercise-induced oxidative stress^2,3,5–7^.

In this study, we employed SFN to activate the Nrf2-ARE pathway in mice. We assessed effects of Nrf2 activation on exercise endurance capacity by estimating running distances of mice subjected to an exhaustive (progressive-continuous all-out) treadmill test. The expression of markers for oxidative stress and tissue damage were then measured in mouse muscles.

## Materials and Methods

### Animals and Treatments

The present study was conducted in accordance with the principles and guidelines for international animal care and was approved by the Institutional Animal Care and Use Committees of the University of Tsukuba (Ibaraki, Japan).

Twelve- to 13-week-old (body weight, 25–28 g) male wild-type mice (presence of Nrf2; *Nrf2* ^+/+^) and Nrf2-null mice (absence of Nrf2; *Nrf2* ^−/−^) on C57BL/6J background were housed in a colony cage under a 12 h:12 h light-dark cycle at 22.5 ± 1.4 °C and 55.6 ± 4.0% relative humidity. Mice were provided with a pellet rodent diet and water ad libitum.

**Figure 1:**
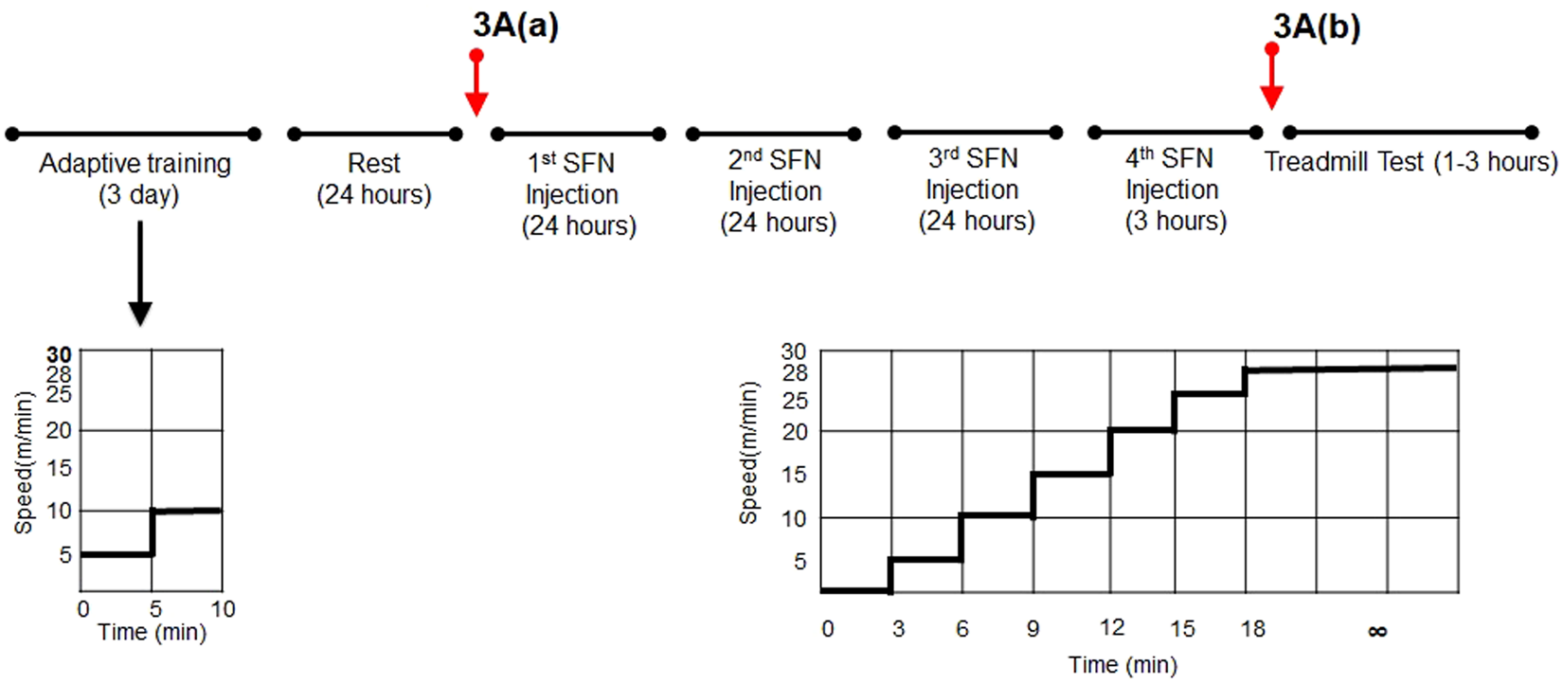
Experimental protocol. For 3 days, all mice performed 10 min of adaptive training at low speed for 5–10 m/min. Then, all mice were injected with SFN or vehicle four times for 3 days (72, 48, 24, and 3 h prior the exhaustive treadmill tests). On the day of the test, all mice conducted the experimental protocol. Exercise intensity was incrementally increased from running with the treadmill set at an initial speed of 5 m/min to a maximum speed of 28 m/min every 3 min. The maximal velocity was maintained until mice were exhausted. Red arrows indicate the time course of *in vivo* imaging after SFN or vehicle injection. Results are presented in Fig. 3A[(a)~(b)].

Subject mice of both genotypes (*Nrf2* ^+/+^ and *Nrf2* ^−/−^) were randomly divided into the following four groups of eight mice each: 1) *Nrf2* ^*+/+*^ mice pretreated with sulforaphane (SFN) (*Nrf2* ^*+/+*^ [SFN]); 2) *Nrf2* ^*+/+*^ mice pretreated with vehicle (*Nrf2* ^*+/+*^ [CON]); 3) *Nrf2* ^−/−^ mice pretreated with SFN (*Nrf2* ^−/−^ [SFN]); and 4) *Nrf2* ^−/−^ mice pretreated with vehicle (*Nrf2* ^−/−^ [CON]). Mice were injected with SFN or vehicle four times for 3 days (72, 48, 24, and 3 h prior to the exhaustive treadmill tests). The injection frequency and timing of SFN injection was based on results from preliminary experiments to determine optimum Nrf2 activation. *Nrf2* ^*+/+*^ [SFN] and *Nrf2* ^−/−^ [SFN] mice were intraperitoneally injected with SFN [25 mg/kg^12^ (MPBio, CA)]. SFN was solubilized in 100% dimethyl sulfoxide (Nacalai Tesque Inc.), and the stock solution was diluted with phosphate-buffered saline. *Nrf2* ^*+/+*^ [CON] and *Nrf2* ^−/−^ [CON] mice were injected with equal amounts of dimethyl sulfoxide dissolved in phosphate-buffered saline (Fig. 1).

### Sample collection

All mice were sacrificed by cervical dislocation under diethylether anaesthesia at baseline, at 50 min following initiation of the exhaustive treadmill test, immediately upon completion of the test, and 18 h following completion of the test. Quadriceps, gastrocnemius, and soleus muscles on the animals’ right side were dissected. Blood was collected from the abdominal aorta and centrifuged (3000 rpm for 20 min at 4 °C) to isolate serum. Samples were frozen in liquid nitrogen and stored at −80 °C pending analysis.

### Antibodies and immunoblot analyses

Antibodies were obtained from the following sources: Peroxisome proliferator-activated receptor gamma coactivator 1-alpha (PGC-1α; Abcam Cat. #ab54481); AMP-activated protein kinase α (AMPKα; Cell Signaling Cat. # 2532); phosphonate-AMPKα (Cell Signaling Cat. # 2535); Sirtuins 1 (SirT1; Cell Signaling Cat. # 2310); α-tubulin (Cell Signaling Cat. # 3873); and Lamin A/C (Cell Signaling Cat. # 4777).

For western blot analysis, right gastrocnemius muscle lysates were separated by SDS-PAGE and transferred onto nitrocellulose membrane (Bio-Rad, Mississauga, Canada). The membrane was incubated in blocking buffer for one hour (Blocking one-P; Nacalai Tesque Inc.), followed by incubation overnight at 4 °C with the primary antibodies. Then, the blot was incubated with HRP-conjugated secondary antibodies for 2 hours at room temperature and signals were visualized with enhanced chemiluminescence (Chemi-Lumi One Super; Nacalai Tesque Inc.). Target proteins was exposed using by a ChemiDoc XRS + system chemiluminescence imager (Bio-Rad, Hercules, CA, USA). Image Lab software (Bio-Rad) was used for image acquisition and densitometry analysis.

Nuclear protein was extracted from right gastrocnemius muscle using a NE-PER^™^ Nuclear and Cytoplasmic Extraction Reagent Kit (Thermo Fisher Scientific, MA, USA), according to the kit protocol. Nuclear samples were analysed by SDS-PAGE and western blotting as described above.

### Histochemical analysis

Right gastrocnemius muscles were placed in isopentane (C_5_H_12_; Nacalai Tesque Inc.) and frozen in liquid nitrogen. A serial cross-section (10 µm) of the muscle was cut transversely using a cryostat (CM 300; Leica Japan, Tokyo, Japan) at −20 °C and stained for myosin-ATPase (preincubation: pH 10.8). Section images were captured using a photomicrographic camera (Olympus AX70; Olympus, Tokyo, Japan) attached to a light microscope (Olympus BX51; Olympus). Images were processed and randomly calculated for percent fibre type (Type I stained light and Type II stained dark) and for cross-sectional areas, using image analysis software (ImageJ; National Institutes of Health, Bethesda, MD, USA)

### RNA isolation and reverse transcription

Right gastrocnemius and soleus muscle tissues (20 mg) were homogenized in 1 mL Sepasol-RNA I Super G (Nacalai Tesque Inc.). RNA was isolated via chloroform extraction and precipitated with 2-propanol. RNA was then washed with 75% ethanol and re-dissolved in RNAse-free water. RNA quality and quantity were determined using a spectrophotometer (NanoVue; GE Healthcare, Uppsala, Sweden). First-strand cDNA synthesis was performed using a PrimeScript RT reagent kit (Takara Bio Inc., Shiga, Japan) following the kit protocol.

### Mitochondrial DNA Copy Number

Genomic DNA in right gastrocnemius muscle tissues was extracted using a Qiagen DNA Genomic-tip kit according to the kit protocol. Using the DNA, mitochondrial copy number was estimated by real-time quantitative polymerase chain reaction as described below from the respective quantities of nuclear and mitochondrial DNA, which we considered to be the concentration of mitochondria per cell in the tissue.

The following primers (Fasmac, Tokyo, Japan) were used in this assessment: Mitochondrial DNA [mtDNA; F, 5′-CCCTAAAACCCGCC ACATCT-3′, R, 5′-GAGCGATGGTGAGAGC TAAGGT-3′], nuclear DNA [nDNA; F, 5′-CGAGTCGTCTTT CTCCTGATGAT-3′, R, 5′-TTCTGGATTCCAA TGCTTCGA-3′].

### Real-time quantitative polymerase chain reaction

Two microliters of the DNAs (diluted to 1:10) were added to Fast SYBR Green Master Mix (Applied Biosystems, Santa Ana, CA). Polymerase chain reaction was performed in 20 µL reaction volumes on a CFX384 Touch Real-Time PCR Detection System (Bio-Rad Lab, CA, US). The following PCR cycling conditions were used for all primer pairs: initial enzyme activation at 95 °C for 20 s followed by 40 cycles of denaturation at 95 °C for 1 s and annealing at 60 °C for 20 s. Fluorescence data were collected at the end of the extension step. After cycling, the melting curve was determined in the range of 65–95 °C with temperature increments of 0.1 °C/s. Each reaction was run in triplicate with appropriate negative controls. Data analysis was performed using the delta-CT-method.

The following primers (Fasmac) were used in this study: haeme oxygenase-1 [HO-1; Forward (F), 5′-CCTCACTGGCAGGAAATCATC-3′, Reverse (R), 5′-CCTCGTGGAGACGCTTTACATA-3′], NAD (P) H: quinone oxidoreductase NQO1; [F, 5′-GGGTCGTCTTGGCAACCA-3′, R, 5′-CAGATGTTGAGGGAGGATCGTAA-3′], γ-glutamylcysteine synthetase [γ-GCS; F, 5′-AGAAGGGGGAGAGGACAAAC-3′, R, 5′-AGTGATGGTGCAGAGAGCCT-3′], and catalase [F, 5′-GGTCACCCACGATATATCACCAGATAC-3′, R, 5′-CGAGGGTCACGAACTGTGTCA-3′], nuclear respiratory factor 1 [NRF-1; F, 5′-GAACGCCACCGATTTCACTGTC-3′, R,5′-CCCTACCACCCACGAATCTGG-3′], transcription factor A, mitochondrial [TFAM; F,5′-CTGATGGGTATGGAGAAGGAGG-3′, R, 5’-CCAACTTCAGCCATCTGCTCTTC-3′], p53R2 [F, 5′-CCAGGTTACCATGGTTGTGG-3′, R, 5′-CCAGTGCACTCAGTAGCTGTG-3′], cytochrome c oxidase [COX IV; F, 5′-ACCAAGCGAATGCTGGACAT-3′, R, 5′-GGCGGAGAAGCCCTGAA-3′], SCO1 [F,5′-CTAGCTTAGCACAATAGCAAGGGCAGGCTAC-3′, R,5′-CCCAGGAATGCAGTTATGACATGACAGCAAAGGCA-3′

SCO2[F, 5′-CAGCCTGTCTTCATCACTGTGGA-3′, R, 5′-GACACTGTGGAAGGCAGCTATGTGCC-3′]. Data were normalized against GAPDH [F, 5′-GTCTTCACCACCATGGAGAAGGCT-3′ and R, 5′-CATGCCAGTGAGCTTCCCGTTCA-3′] and expressed as mean values.

### Biochemical analysis

Serum levels of lactate dehydrogenase (LDH) and creatine phosphokinase (CPK) in serum samples were analysed based on the Japan Society of Clinical Chemistry transferable method. Free fatty acids (FFAs) were analysed using the enzymatic method of Kotobiken Co. Ltd. (Tokyo, Japan). Blood samples were obtained by nicking the lateral tail vein using sharp surgical scissors. Blood glucose level was measured using a hand-held glucometer (Ascensia Breeze^®^2; Bayer Health Care, Osaka, Japan). Blood lactate level was determined using a hand-held lactate analyser (Lactate Pro; Arkray KDK, Kyoto, Japan). Blood was sampled through the tail vein. A commercial enzyme-linked immunosorbent assay kit was used to measure levels of thiobarbituric acid reactive substances (TBARS; Cayman Chemical, MI) from homogenates of gastrocnemius muscles. Reduced (GSH) and oxidized (GSSG) glutathione levels of gastrocnemius muscles were assayed using a GSH/GSSG Ratio Detection Assay Kit (Fluorometric, Abcam, Cambridge, UK).

Adenosine triphosphate (ATP) levels in right quadriceps muscles were measured using a luciferin-luciferase assay kit (TOYO Ink, Tokyo, Japan.). Glycogen content in the quadriceps muscles was measured using a Glycogen Assay kit (Biovision, CA). The level of cyclic AMP (cAMP) in gastrocnemius muscle was measured using a cAMP Direct Immunoassay Detection Kit (Fluorometric, Abcam, Cambridge, UK).

### Exhaustive (progressive-continuous all-out) treadmill test

A motor-driven two-lane treadmill chamber was used during the exhaustive treadmill tests to analyse respiratory parameters such as oxygen (VO_2_) consumption, carbon dioxide (VCO_2_) production, and respiratory quotient (RQ) (Muromachi, Tokyo, Japan). Fresh air was pumped into the chamber at a flow rate of 1.5 L/min during the tests.

For 3 days prior to injection with SFN or vehicle, all mice were subjected to 10 min adaptive training at low speed (5–10 m/min) to familiarize them with the treadmill. During the test, all mice were placed on a horizontal treadmill, after which exercise intensity was incrementally increased from an initial running speed of 5 m/min to a maximum speed of 28 m/min every 3 min; maximal velocity was maintained until mice were exhausted (see below for exhaustion criteria) (Fig. 1). All experimental protocols were performed during the mouse dark cycle.

The criterion for exhaustion was defined as the point at which the mice failed to run on the treadmill even after hand prodding using a soft bristle brush and electrical grid and failure of the righting reflex when the mice were laid on their backs.

### Indirect calorimetry

Metabolic parameters were measured using an indirect calorimeter (Muromachi) at the Laboratory Animal Resource Center (University of Tsukuba). Calorimetry measurements were acquired during a 2-day acclimation period at baseline. VO_2_ and VCO_2_ levels were measured in each chamber every 3 min. The flow rate was set at 0.6 L/min during the measurements. Energy expenditure (EE) was calculated as VO_2_ × [3.815 + (1.232 × RQ)]^13^.

### Nrf2-Luciferin *in vivo* imaging and luciferase activity detection

Transgenic OKD48 (Keap1-dependent oxidative stress detector, No-48-luciferase) mice were used for Nrf2 activity detection using *in vivo* imaging^14^. We used mice produced by crossing an OKD48 mouse with an Nrf2^+/+^ (albino-BL/6) or Nrf2^−/−^ (albino-Nrf2^−/−^) mouse to improve signal detection. After intraperitoneal injection with 150 mg/kg d-luciferin (Promega, Madison, WI, USA) dissolved in PBS, mice were imaged using an *In Vivo* Imaging System (Vivo Vision IVIS Spectrum; Xenogen, Alameda, CA, USA) under anaesthesia with isoflurane. At 2 min following injection, mice were placed in the *In Vivo* Imaging System imaging chamber. Data were collected using high sensitivity, 10 min exposure, followed by analysis with Living Image software (Xenogen).

### Luciferase reporter assays

We used the Luciferase Assay System (Promega, Madison, WI, USA). Briefly, 20 mg of the right gastrocnemius muscle containing the firefly luciferase gene of the OKD48 mouse was homogenized in Passive Lysis Buffer. After incubation at 4 °C for 3 h, homogenates were centrifuged at 12,000 rpm for 5 min. Supernatants (20 μL) were centrifuged and mixed with 100 μL of luciferase assay reagent, after which total light emission was measured using a TD-20/20 luminometer (Turner Design, CA, USA) according to the manufacturer’s protocol. The total protein in each supernatant was measured using the Pierce BCA protein assay kit (Thermo Fisher Scientific). Luciferase activities were calculated per mg protein in the gastrocnemius muscle.

### Statistics

Statistical analysis was performed using SPSS Statistics for Windows, version 23.0 (IBM Corporation, Armonk, NY, USA). Descriptive parameters were expressed as mean ± SE. To compare between groups for all dependent variables, we performed analysis with a one-way analysis of variance. *P* < 0.05 was considered significant.

## Results

### Baseline assessment

**Figure 2:**
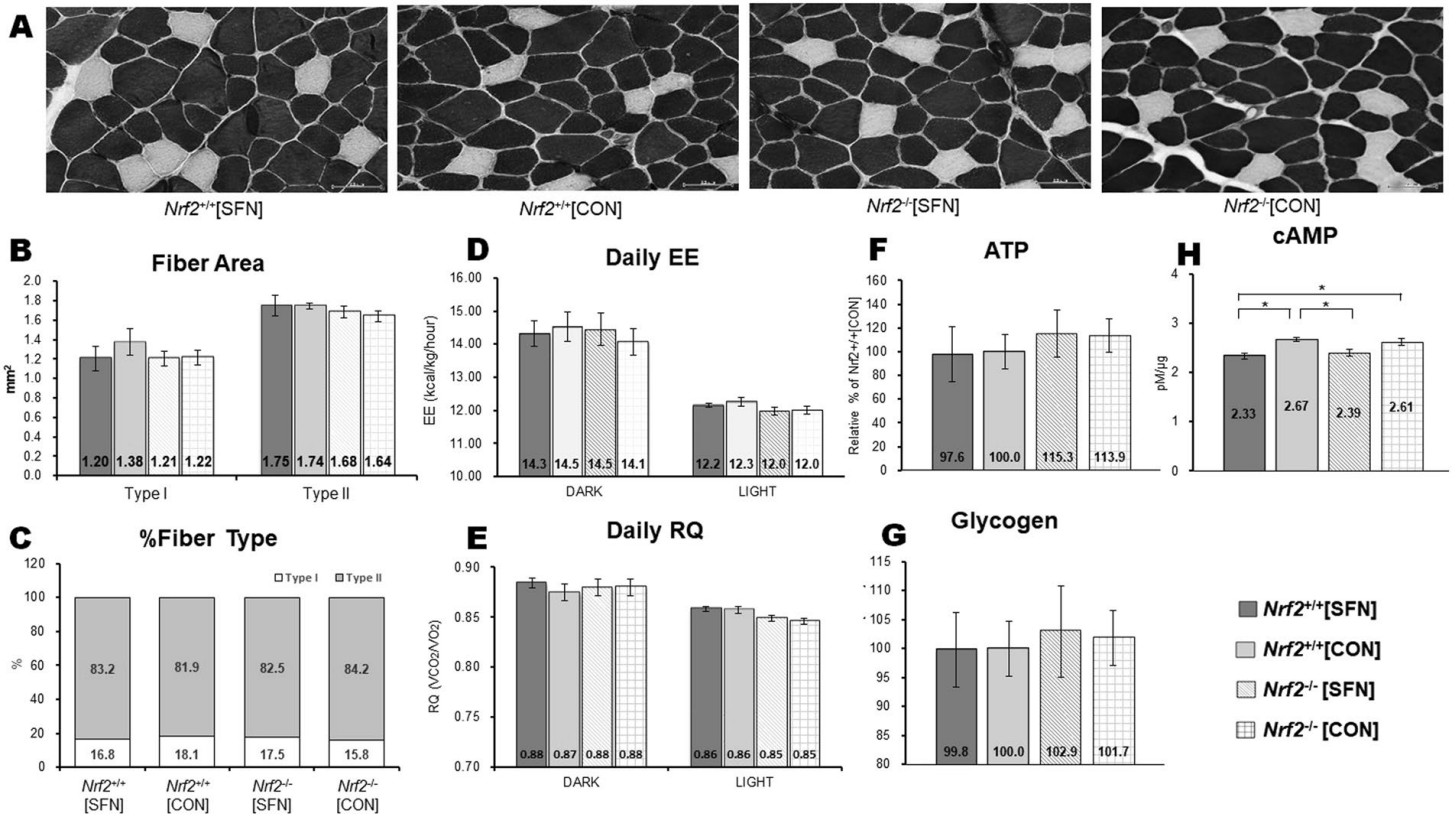
Fibre types in gastrocnemius muscle obtained by ATPase staining at pH 10.8 following SFN or vehicle injection (**A**). Fibre area (**B**) and fibre-type distribution (**C**) in the gastrocnemius muscle were determined by ATPase staining. Daily EE (**D**) and RQ (**E**) values assessed during each dark and light cycle using an indirect calorimeter. ATP (**F**), Glycogen (**G**) and cAMP (**H**) levels were measured in gastrocnemius muscle.

ATPase-stained cross sections of muscle fibres from the central portion of the deep gastrocnemius of the lower right limb were obtained from *Nrf2* ^*+/+*^ and *Nrf2* ^−/−^ mice injected with SFN or vehicle (Fig. 2A). Diameters of Type I and Type II fibres were not significantly different in *Nrf2* ^+/+^ and *Nrf2* ^−/−^ mice. Fibre diameters did not change following four SFN injections (Fig. 2B). There were no significant differences in the ratio of Type I to Type II muscle fibres between *Nrf2* ^+/+^ and *Nrf2* ^−/−^ mice. These ratios were not significantly altered following four SFN injections (Fig. 2C). There were no significant differences in energy expenditure (Fig. 2D) and respiratory quotient (RQ) (Fig. 2E) for each dark and light cycle among all groups. There were no significant differences in mean levels of ATP (Fig. 2F), glycogen (Fig. 2G), and cAMP (Fig. 2H) among all groups. These data suggest that Nrf2 genotype and SFN injection did not significantly affect muscle fibre morphology, physiology, or metabolism at baseline.

### Mitochondria biogenesis

**Figure 3:**
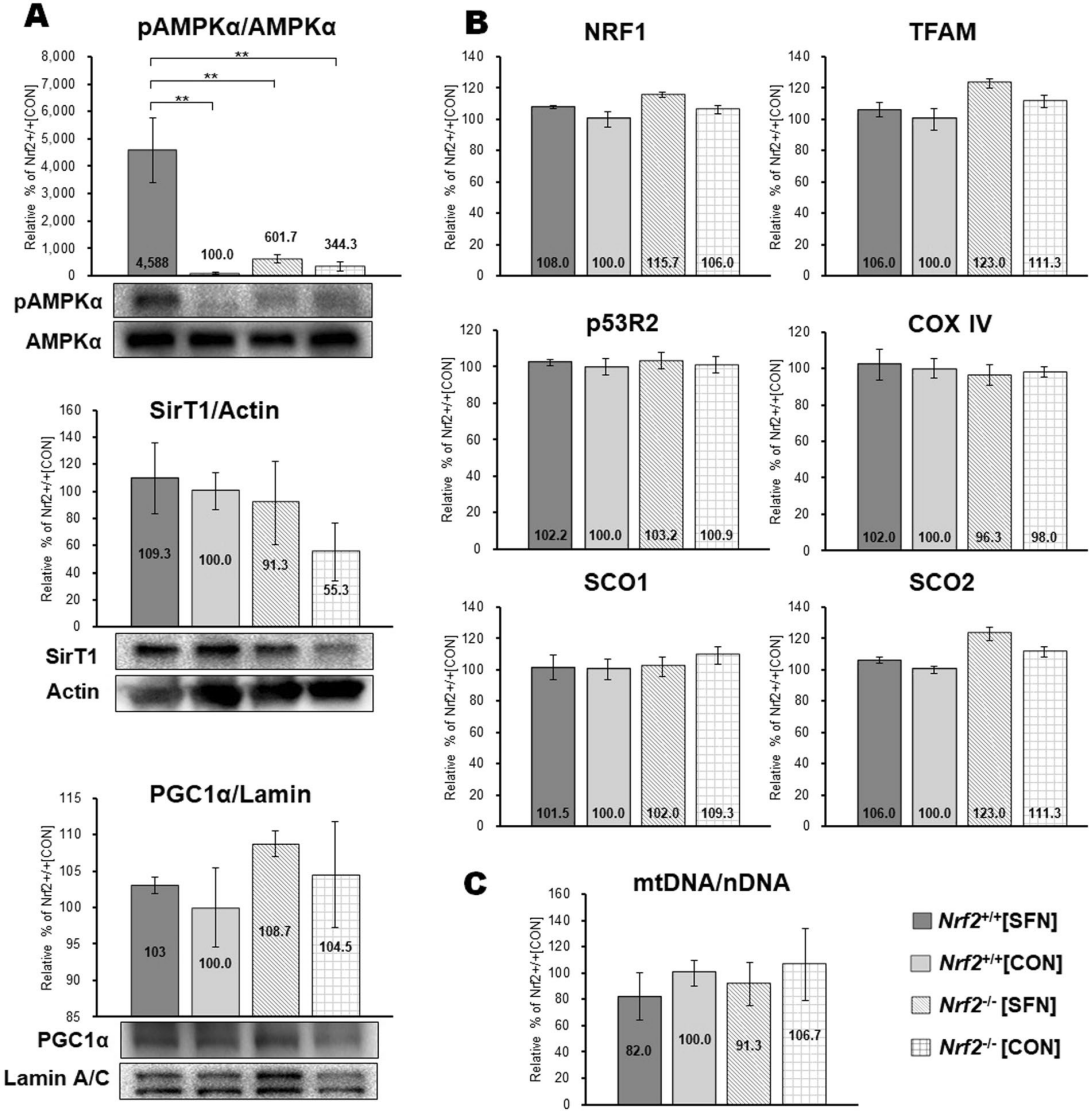
Effect of SFN injection. SFN or vehicle was injected four times for 3 days and mitochondrial biogenesis markers were measured in mice gastrocnemius muscle. (**A**) protein expression levels (western blot strips) of mitochondrial biogenesis markers. The amount of phosphorylated AMPKα was normalized to the amount of AMPKα protein. Protein content of mitochondrial biogenesis markers SirT1 and PGC1α were also measured. The loading volume was normalized by the expression levels of actin and lamin A/C, respectively. (**B**) mRNA expression of mitochondrial biogenesis markers in AA. mRNA levels of NRF1, TFAM, p53R2, COX IV, SCO1, and SCO2 were determined by real-time PCR. Values were normalized to the level of the housekeeping gene, GAPDH. (**C**) mtDNA copy number in the gastrocnemius muscle. This number factors into the ratio of nuclear and mtDNA (mtDNA/nDNA); brackets **P < 0.01.

Figure 3 displays biomarkers for mitochondria biogenesis in gastrocnemius homogenates at baseline. Western blot analysis revealed that phosphorylated AMPKα significantly increased in *Nrf2* ^+/+^[SFN] mice following four SFN injections (Fig. 3A). However, there were no significant differences in protein expression of SirT1 and PGC1α among all groups. Figure 3B shows mRNA expression of mitochondrial biogenesis markers, including NRF-1, TFAM, p53R2, COX IV, SCO1 and SCO2. No elevated mRNA expression was detected in any group. There was an elevated mtDNA copy number relative to nDNA. This is considered a good marker for mitochondrial biogenesis^15^. After the fourth SFN injection in *Nrf2* ^*+/+*^ mice and in Nrf2 in *Nrf2* ^−/−^ groups, mtDNA copy number was not significantly different (Fig. 3C). These data suggest that the connection was weak between activating mitochondria biogenesis signaling and the SFN injection, which was administered only four times.

### Luminescence activity

**Figure 4:**
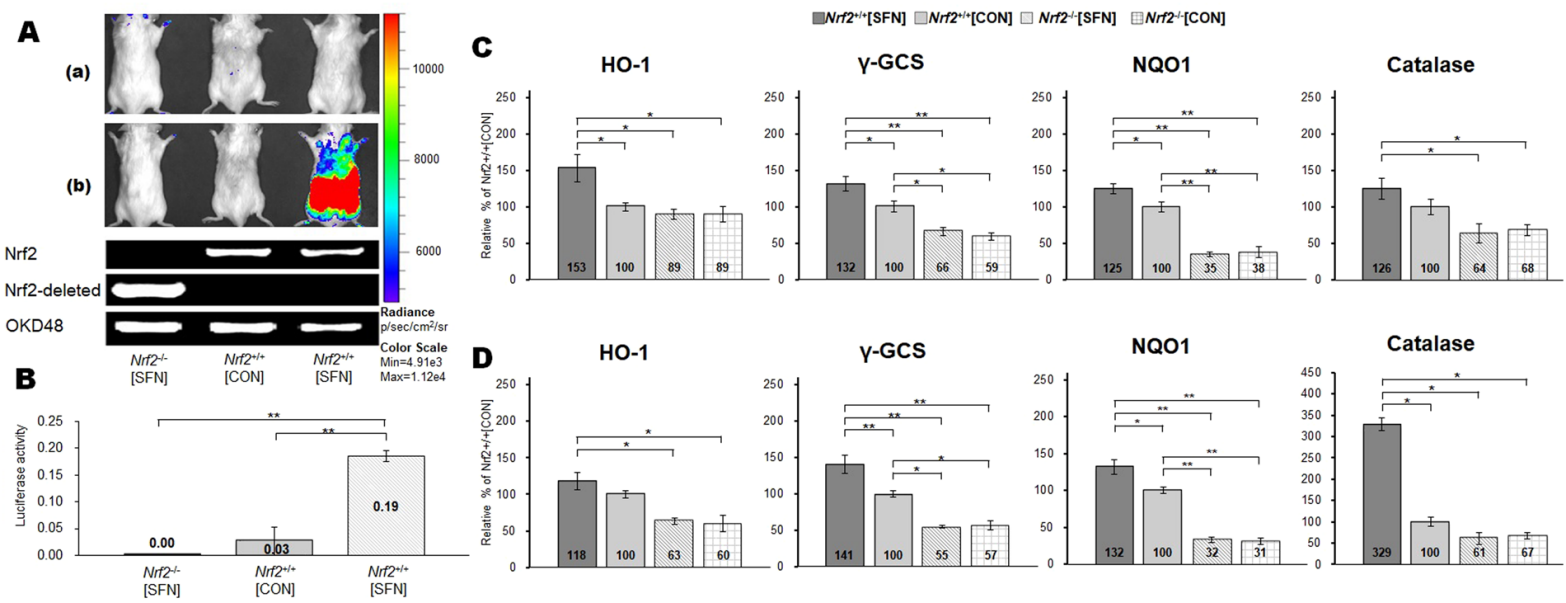
SFN-induced Nrf2-Luc activity detected using an *In Vivo* Imaging System and results of genotyping of Nrf2 and OKD48 transgenic mice (**A**) in the prone position. Time course [(a: baseline) ~ (b: following SFN injections)] of luciferase assay is shown in Fig. 1. Nrf2-Luc activity in gastrocnemius muscle after the fourth SFN injection measured using a luminometer (**B**). mRNA levels of Nrf2 target genes in gastrocnemius (**C**) and soleus (**D**) muscles following four injections of SFN or vehicle. Brackets *P < 0.05, **P < 0.01.

Figure 4A shows real-time *in vivo* imaging of an Nrf2 response during SFN injection in transgenic OKD48 (Keap1-dependent oxidative stress detector, No-48-luciferase) mice. The OKD48 mice expressed the N-terminal half of Nrf2-Luc fusion protein, driven by the Nrf2-based 3xAre promoter^13^. No Nrf2-Luc activation was detected in *Nrf2* ^+/+^[CON] and *Nrf2* ^−/−^[CON] mice. Substantial Nrf2 upregulation was observed in *Nrf2* ^+/+^[SFN] mice 3 h following the fourth SFN injection (at 75 h), shortly before the exhaustion treadmill test [Fig. 4A(a) and (b)]. During the SFN injection period, Nrf2-Luc activation gradually increased in *Nrf2* ^+/+^[SFN] mice at baseline at 24, 48, 72, and 75 h [Fig. S1(a),(b),(c),(d), and (e)]. Nrf2-Luc was not activated in the *Nrf2* ^+/+^[CON] and *Nrf2* ^−/−^[CON] groups. Luciferase activity of the gastrocnemius muscle is shown in Fig. 4B. Nrf2-Luc was upregulated in the muscles of *Nrf2* ^+/+^[SFN] mice at 75 h. This change was significantly higher than those of the other groups (*P* < 0.01). These results suggest that SFN pretreatment upregulates Nrf2.

### Nrf2 target genes

Figure 4C,D display the mRNA levels of selected Nrf2 target genes: haeme oxygenase-1 (HO-1), NAD (P) H: quinone oxidoreductase A (NQO1), gamma-glutamylcysteine synthetase (γ-GCS), and catalase. These levels were measured in gastrocnemius (Fig. 4C) and soleus (Fig. 4D) tissue homogenates with and without SFN pretreatment. *Nrf2* ^+/+^[SFN] mice demonstrated significantly higher expression levels of all target genes in both the gastrocnemius and soleus muscles compared with *Nrf2* ^−/−^ groups (*Nrf2* ^−/−^ [SFN] and *Nrf2* ^−/−^[CON]). Between the two *Nrf2* ^+/+^groups, catalase was not upregulated in the gastrocnemius. HO-1 was not upregulated in the soleus in *Nrf2* ^+/+^[SFN] mice than those in *Nrf2* ^+/+^[CON] mice. However, expression levels of the other Nrf2 target genes were significantly higher in both tissues in *Nrf2* ^+/+^[SFN] mice compared with those in *Nrf2* ^+/+^[CON]: NQO1 and γ-GCS expression levels in gastrocnemius and soleus of *Nrf2* ^+/+^[CON] mice were significantly higher than those in *Nrf2* ^−/−^ mice.

### Exercise endurance capacity and energy metabolism parameters

**Figure 5:**
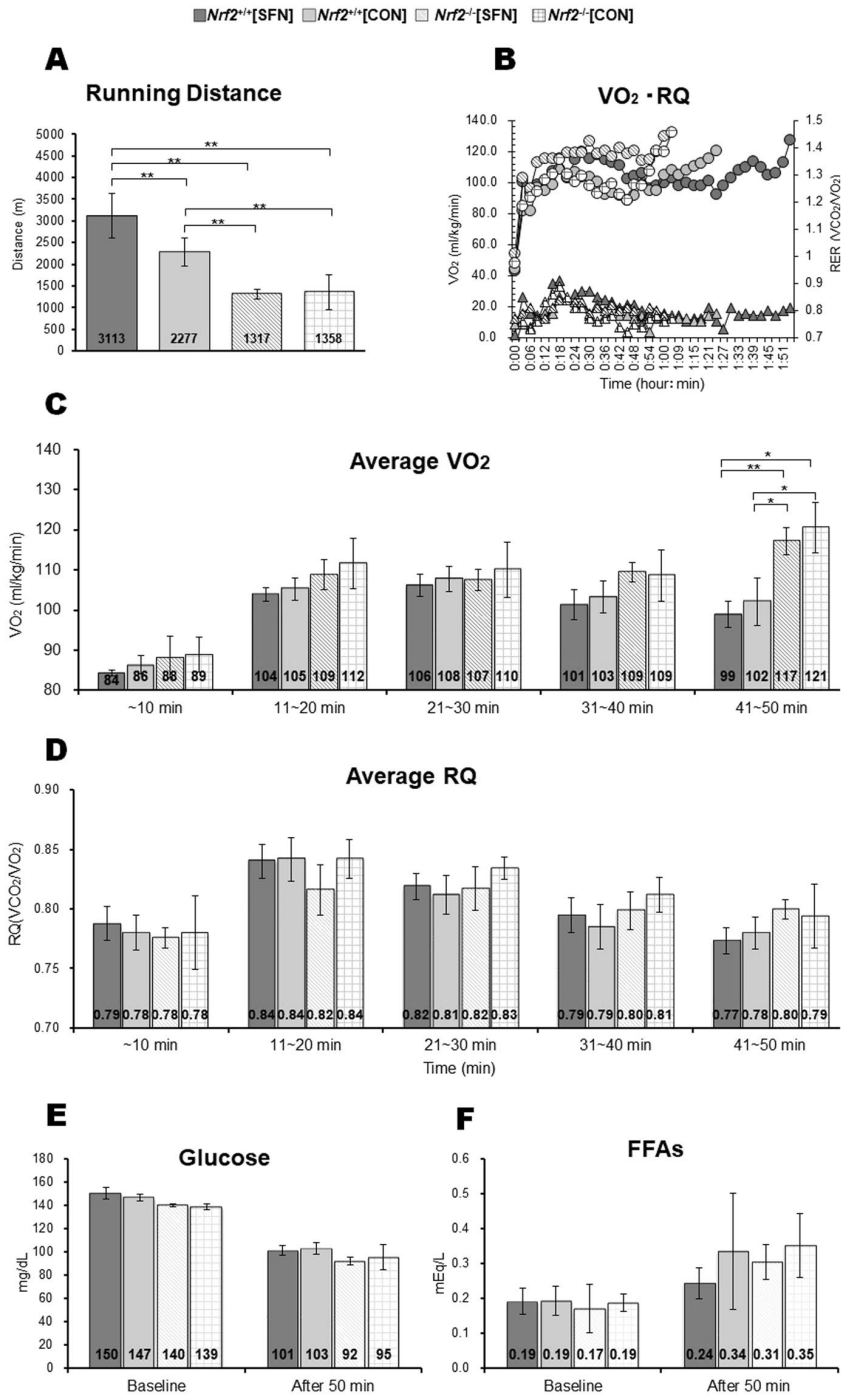
Running distance of each group in exhaustive treadmill test (**A**) and VO_2_ (circle) and RQ (triangle) per running time in mouse recorded median running distance in each group (**B**). Average levels of energy substrates: VO_2_ (**C**), RQ (**D**), glucose (**E**), and FFA (**F**) in each group after the 50-min exhaustive treadmill test for comparison among groups at the same time course. Brackets *P < 0.05, **P < 0.01.

Figure 5A shows the results of the running distance test, which was measured as a proxy for exercise endurance capacity. *Nrf2* ^+/+^ mice achieved greater running distances than *Nrf2* ^−/−^ mice. Among *Nrf2* ^+/+^ groups, *Nrf2* ^+/+^[SFN] mice achieved greater running distances than those of *Nrf2* ^+/+^[CON] mice. Figure 5B shows oxygen (VO_2_) consumption and RQ per running time, and the recorded median running distance for each group. Figure 5C and D show average VO_2_ and RQ levels for each group during the 50-min exhaustive treadmill test. There were no significant differences in VO_2_ levels after 40 min of exercise among all groups. From the period between 41 and 50 min, VO_2_ levels in the *Nrf2* ^−/−^ groups were higher than those in the *Nrf2* ^+/+^ groups. However, there were no significant differences among the four groups in terms of average RQ per time period. Serum levels of glucose and free fatty acids (FFAs) showed no significant difference among the four groups at baseline and after 50 min (Fig. 5E and F).

### Biomarkers for oxidative stress and muscle damage

**Figure 6:**
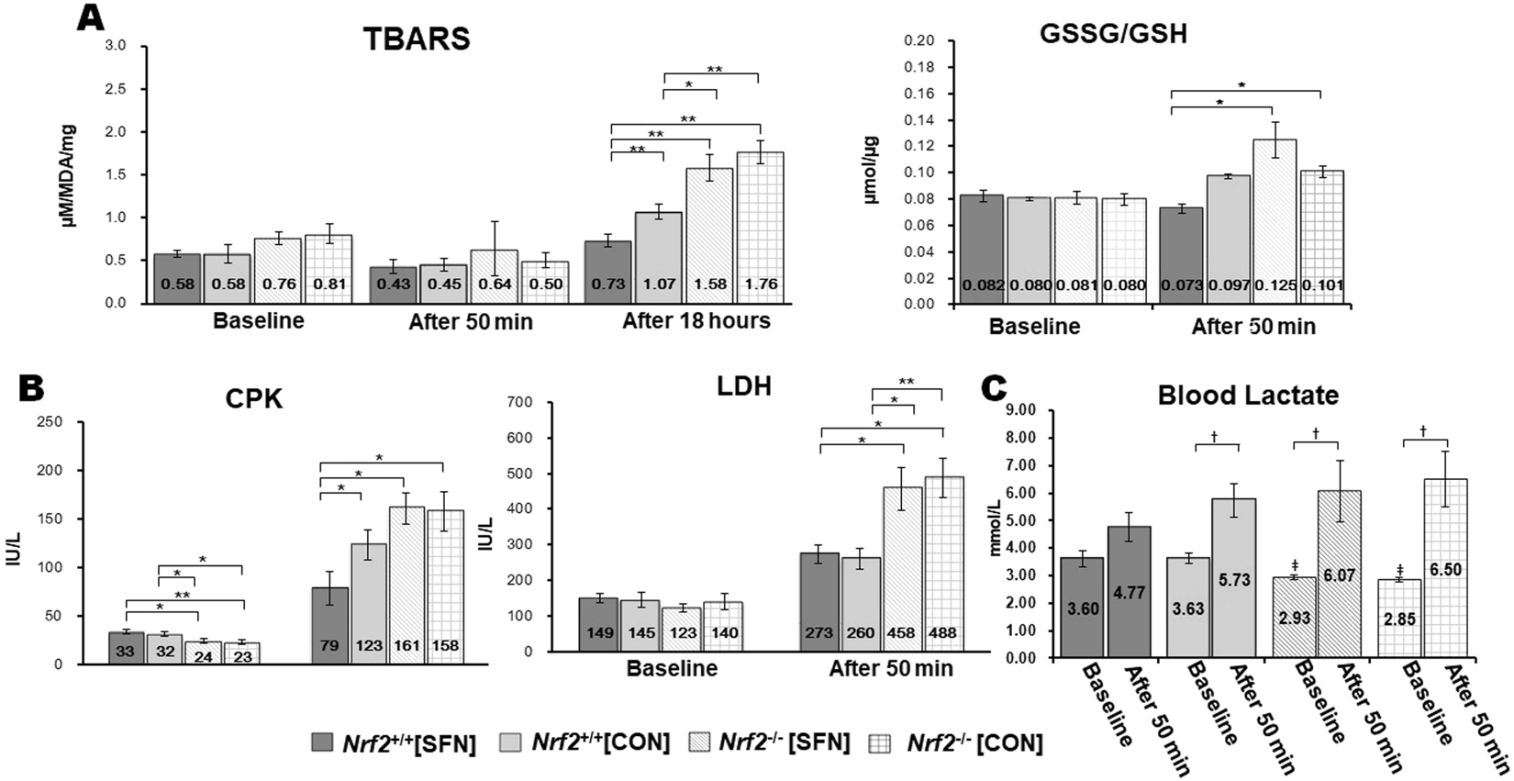
Oxidative, TBARS and GSSG/GSH (**A**) markers in the gastrocnemius muscle, and muscle damage, CPK and LDH (**B**) markers, muscle fatigue, lactate (**C**) marker in the blood. Brackets *P < 0.05, **P < 0.01, ^†^ *P* < 0.05 vs. after the 50-min exhaustive treadmill test, ^‡^ *P* < 0.05 vs. Nrf2^+/+^ groups.

Figure 6A shows levels of thiobarbituric acid reactive substances (TBARS) in gastrocnemius homogenates at baseline, following an exhaustive test at 50 min, and at 18 h following the end of the test. In the baseline and 50-min periods, all groups showed comparable TBARS levels. However, at 18 h following the test, *Nrf2* ^+/+^ mice exhibited significantly lower TBARS levels than those of *Nrf2* ^−/−^ mice. TBARS levels in SFN-injected *Nrf2* ^+/+^ mice were lower than those of uninjected *Nrf2* ^+/+^ mice. For the GSSG/GSH ratio in baseline, all groups also showed to be comparable. However, at 50-min following the test, SFN injection in *Nrf2* ^+/+^ mice showed that the GSSG/GSH ratio was lower than that in *Nrf2* ^−/−^ groups (Fig. 6A). Figure 6B shows serum levels of muscle damage biomarkers creatine phosphokinase (CPK) and lactate dehydrogenase (LDH) for each group at baseline and following 50 min of treadmill testing. CPK levels were significantly higher in *Nrf2* ^+/+^ groups than in *Nrf2* ^−/−^ groups at baseline. At the 50-min mark of exhaustive treadmill testing, CPK levels in *Nrf2* ^+/+^ groups were lower than those in *Nrf2* ^−/−^ groups. In particular, CPK levels in *Nrf2* ^+/+^[SFN] mice were significantly lower compared with other treatment groups. All groups showed comparable LDH levels at baseline. However, LDH levels in *Nrf2* ^+/+^ groups were lower than those in *Nrf2* ^−/−^ groups following the 50-min treadmill test. Blood lactate levels in *Nrf2* ^−/−^ were significantly lower than those of the *Nrf2* ^+/+^ at the baseline. We did not see a significant increase in blood lactate levels in *Nrf2* ^+/+^ mice with SFN injection after 50 minutes. However, other groups showed a significant increase (Fig. 6C).

These data suggest that SFN injection in *Nrf2* ^+/+^ mice exerted protective effects in muscle under exhaustive exercise conditions.

## Discussion

Elevated oxygen utilization in muscles with increasing exercise intensity may promote production of free radicals and other ROS from spill-over reactions of electrons in the mitochondrial electron transport chain^5^. Enhanced free radical formation in active muscles regulates various cellular signalling pathways that may result in restricted muscle contraction^16^. These processes may be caused by impaired central nervous system function, disordered sarcolemmal function, microvascular regulation, calcium regulation, impaired myofilament contraction, and/or altered mitochondrial metabolism^17^. Although controversial^18,19^, a growing body of evidence suggests that nutritional supplementation with molecules that enhance the body’s antioxidant defence system, and that act as ROS scavengers, might prevent exercise-induced oxidative stress and reduce muscle damage^20–22^. Considering the activation of Nrf2, SFN is one such powerful antioxidant^11^.

Our study suggests that SFN pretreatment may increase running distance in exhaustive treadmill tests (Fig. 5A) by suppressing production of TBARS and GSSG-to-GSH ratio in skeletal muscles (Fig. 6A), and by suppressing release of CPK, LDH (Fig. 6B) and lactate (Fig. 6C) in the blood. Increased TBARS levels indicate increased *in vivo* lipid peroxidation. Serum levels of CPK and LDH are often used as measures of structural damage to muscle cells. The accumulation of lactate causes fatigue in muscles. To the best of our knowledge, our study provides the first direct evidence demonstrating that upregulation of the Nrf2 signalling pathway by SFN, as well as its regulation of downstream phase II and antioxidant genes (HO-1, NQO1, γ-GCS, and catalase), play a crucial role in protecting against exhaustive exercise-mediated oxidative stress and tissue damage in skeletal muscles. SFN-induced Nrf2 activation in skeletal muscles might play a critical role in improving exercise endurance capacity.

Another major goal of the present study is evaluation of *in vivo* Nrf2 activation via Nrf2-luciferin imaging in whole body and luciferase activity determination in muscles. To date, there have been many unsuccessful attempts at Nrf2 detection in muscle using SDS–PAGE. We had identified Nrf2 *in vitro* activity via electrical stimulation in C2C12 cells (the size of 95–110 kDa band)^23^, but detecting Nrf2 activity in mouse muscle has proved difficult. Some studies targeted a non-specific band in the 55–65 kDa region as Nrf2^24,25^. In 2012, we were the first to develop a transgenic mouse (OKD48) that was used to investigate the Keap1-Nrf2 pathway via an imaging technique that measures luminescence activity^14^. To improve the detection of the Nrf2 signal, we developed a new mouse model by crossing OKD48 with *Nrf2* ^*+/+*^ (albino-BL/6) or *Nrf2* ^−/−^ (albino-*Nrf2* ^−/−^) mice. Using this new model, we demonstrated that SFN activates the Nrf2 signalling pathway in whole body (Fig. 4A), in muscle tissues (Fig. 4B), and strongly protects against oxidative stresses.

TBARS is well-established biological indicators of oxidative stress^26^. The extent of lipid peroxidation reflects key pathological and toxicological states related to oxidative stress^27^. A growing body of evidence suggests that TBARS concentrations are increased in both plasma and tissues as a result of short bouts of exhaustive exercise^28–30^. Glutathione is known as an antioxidant, and has been used as a marker of oxidative stress. Under oxidative stress conditions, there are lower concentrations of GSH and higher concentrations of GSSG, and thus a GSSG:GSH ratio increase^31^. Many studies have demonstrated that the GSSG:GSH ratio increases in response to exercise training in a manner that is highly correlated with lactate: pyruvate ratios^31,32^. High levels of oxidants can cause contractile dysfunction, mitochondrial dysfunction, and muscle atrophy, all of which give rise to muscle weakness and fatigue^3,16,33^. Our data suggest that reduction in TBARS and GSSG:GSH ratio concentrations following the treadmill test suggests protection against muscle damage^12^, mediated by upregulation of antioxidant and detoxifying genes downstream from SFN-induced Nrf2 activation. Our observation of a relationship between SFN pretreatment and inhibition of oxidants is consistent with recent reports. Malaguti *et al*.^12^ reported that *in vivo* levels of oxidative stress increased after exhaustive treadmill tests but were considerably decreased by SFN pretreatment. Angeloni *et al*. demonstrated that SFN significantly increases total *in vitro* antioxidant activity^34^.

Nrf2 may play crucial roles in lipid and glucose metabolism in addition to attenuating oxidative stress^35–37^. Zhang and co-workers^38^ showed that SFN enhances Nrf2 expression and activates the liver kinase B1/AMPK pathway and downstream genes, such as peroxisome proliferator activated receptor γ coactivator-1, phosphorylated acetyl-CoA carboxylase, and carnitine palmitoyl transferase-1. AMPK is known to be regulated in muscles, and acts by facilitating fatty acid uptake and oxidation, glucose uptake, and mitochondrial biogenesis^39,40^. In addition, Uruno *et al*. demonstrated that Nrf2 regulates skeletal muscle glycogen metabolism, particularly glycogen branching enzyme (GBE1) in the context of a maximal incremental treadmill protocol^41^. It may be the case that the observed increase in SFN improves energy metabolism and is responsible for increased running distance in mice. This represents an important subject for further research and requires further experimentation to improve our understanding of the mechanisms by which SFN influences exercise endurance capacity. In this study, VO_2_ and RQ data were obtained by evaluating energy substrate, carbohydrate, and fat utilization using a treadmill chamber. Dramatic changes in VO_2_ levels were observed from 41 to 50 min between *Nrf2* ^*+/+*^ and *Nrf2* ^−/−^ groups (Fig. 5C). This is accompanied by increased LDH and CPK levels in *Nrf2* ^−/−^ groups (Fig. 6B); however, we detected no significant changes in carbohydrate and fat oxidation and RQ (Fig. 5D). We examined serum levels of glucose and FFAs, which serve as major fuel sources in skeletal muscle^42^, but we demonstrated no difference among the four groups. Therefore, severe muscle damage observed in *Nrf2* ^−/−^ groups could be due to lack of Nrf2 expression during the tests rather than by lack of energy supplementation. Strong SFN-mediated induction of total cellular and mitochondrial antioxidants, as well as phase 2 enzymes in *Nrf2* ^*+/+*^ mice might explain the observed enhanced exercise endurance capacity. The change in skeletal muscular oxidative stress levels may account for increased running distance.

Sun *et al*.^43^ reported that SFN treatment (2 mg/kg) for 8 weeks improved muscle function and reduced pathology by exerting protective effects against oxidative stress in dystrophic muscles in muscular dystrophy (mdx) mice. These results suggest that SFN injection of *Nrf2* ^*+/+*^ mice and the absence of Nrf2 in *Nrf2* ^−/−^ groups could affect muscle composition, which in turn could contribute to the observed differences in exercise endurance capacity. However, in our study, injection with SFN over 4 days did not change the muscle fibre types and sizes (Fig. 2A–C). Fibre composition and morphology was not influenced by the presence or absence of Nrf2 genes. Therefore, we propose that SFN-induced improvement in exercise capacity in current exhaustive tests is not mediated by changes in muscle composition.

We displayed markers of mitochondrial biogenesis (Fig. 3) to clarify the cause of the increased exercise capacity. We found significant activation of AMPKα after four SFN injections (Fig. 3A), consistent with a recent study^32^. However, we did not measure a significant difference in other mitochondria biogenesis markers, including mtDNA copy number among the four groups (Fig. 3A–C). In addition, we assessed changes in cAMP (Fig. 2H), thought to be an effect of mitochondria biogenesis^44^. However, we did not measure a significant difference in cAMP among the groups. Kitaoka *et al*.^45^ demonstrate that the presence of Nrf2 gene does not influence the alterations in mitochondrial morphology. Thus, we assume that the connection was weak between activating mitochondria biogenesis signalling and the improvement in exercise endurance capacity through SFN injection, which was administered only four times.

There are inconsistencies with some research^41,46^ reported compared with exercise capacity between Nrf2^+/+^ and Nrf2^−/−^ mice. We recognized that this inconsistency arose from a difference of exercise endurance test protocol. Because the most important goal of this study was to establish a difference in exercise capacity in SFN injection of Nrf2^+/+^ mice and the absence of Nrf2 in Nrf2^−/−^ groups, we put more value on the exercise endurance test protocol. Through a pilot experiment, we found that the result of exercise endurance capacity can vary widely by the exercise test protocols, among which we have chosen the optimal test protocol to demonstrate our hypothesis. The studies mentioned earlier measured the exercise capacity by incremental increase in speed and/or incline to maximal exercise level on a treadmill. There was the possibility of using anaerobic power rather than aerobic power in exercise endurance capacity tests in this study. Provided that it is based on anaerobic power, the leg’s power of the mouse cannot follow the higher speed load and slope resistance. Therefore, the discordance of results between researches following the test protocols is quite thinkable. However, we recognized maintaining the ~28 m/min to all-out is very appropriate for the assessment of exercise endurance capacity.

Recently, there have been important reports^18,19^ related to ROS and exercise adaptation in muscle. That is, antioxidant supplementation might hamper exercise training-induced adaptations in skeletal muscle. Therefore, we should be cautious drawing conclusions regarding long-term SFN treatment. However, even though an antioxidant has beneficial effects, it can have toxic side effects according to type, duration, frequency, and dosage. Therefore, we believe more scientific evidence behind antioxidant functions is needed. We do not consider sulforaphane to be merely an antioxidant. Of course, it is obvious that the main role of Nrf2 activation by sulforaphane treatment is to indirectly express various transcription factors with antioxidant properties. However, it is difficult to state that sulforaphane directly affects other antioxidants to induce cellular signalling for muscle adaptation. Therefore, more experiments are necessary to answer this question to determine long-term negative impacts of SFN.

Our results suggest that SFN-induced Nrf2 upregulation and SFN-mediated antioxidative effects might play crucial roles in attenuating muscle fatigue by reducing oxidative stress caused by exhaustive exercise. These effects may give rise to improved exercise endurance capacity. We believe this study provides new insights into the role of Nrf2 in improving exercise performance.

## Electronic supplementary material

Supplementary Figure
