# Supplementary material for: Nuclear factor (erythroid derived 2)-like 2 activation increases exercise endurance capacity via redox modulation in skeletal muscles
Source: Sci Rep. 2017 Oct 10;7:12902. doi: 10.1038/s41598-017-12926-y (PMC5635018; doi:10.1038/s41598-017-12926-y)
Supplement: Supplementary file 1 — Supplementary Figure [file 41598_2017_12926_MOESM1_ESM.pdf]

Supplementary information

Nuclear factor (erythroid derived 2)-like 2 activation increases exercise endurance capacity via redox modulation in skeletal muscles

Sechang Oh[1,2,5,+], Shoichi Komine[2,+], Eiji Warabi[2], Kentaro Akiyama[2,5], Akiko Ishii[2], Kazunori Ishige[2], Yuji Mizokami[2], Keisuke Kuga[2], Masaki Horie[2], Yoshihiro Miwa[2], Takao Iwawaki[3], Masayuki Yamamoto[4], and Junichi Shoda[1,2,*]

[1]The Center of Sports Medicine and Health Sciences, Tsukuba University Hospital, Tsukuba, Ibaraki 305-8576, Japan;

[2]Faculty of Medicine, University of Tsukuba, Tsukuba, Ibaraki 305-8575, Japan;

[3]Department of Life Science, Medical Research Institute, Kanazawa Medical University, Uchinada, Kahoku, Ishikawa 920-0293, Japan;

[4]Department of Medical Biochemistry, Tohoku University Graduate School of Medicine, Sendai, Miyagi 980-8575, Japan;

[5]Japan Society for the Promotion of Science, Tokyo 102-0083, Japan

[+]These authors contributed equally to this work.

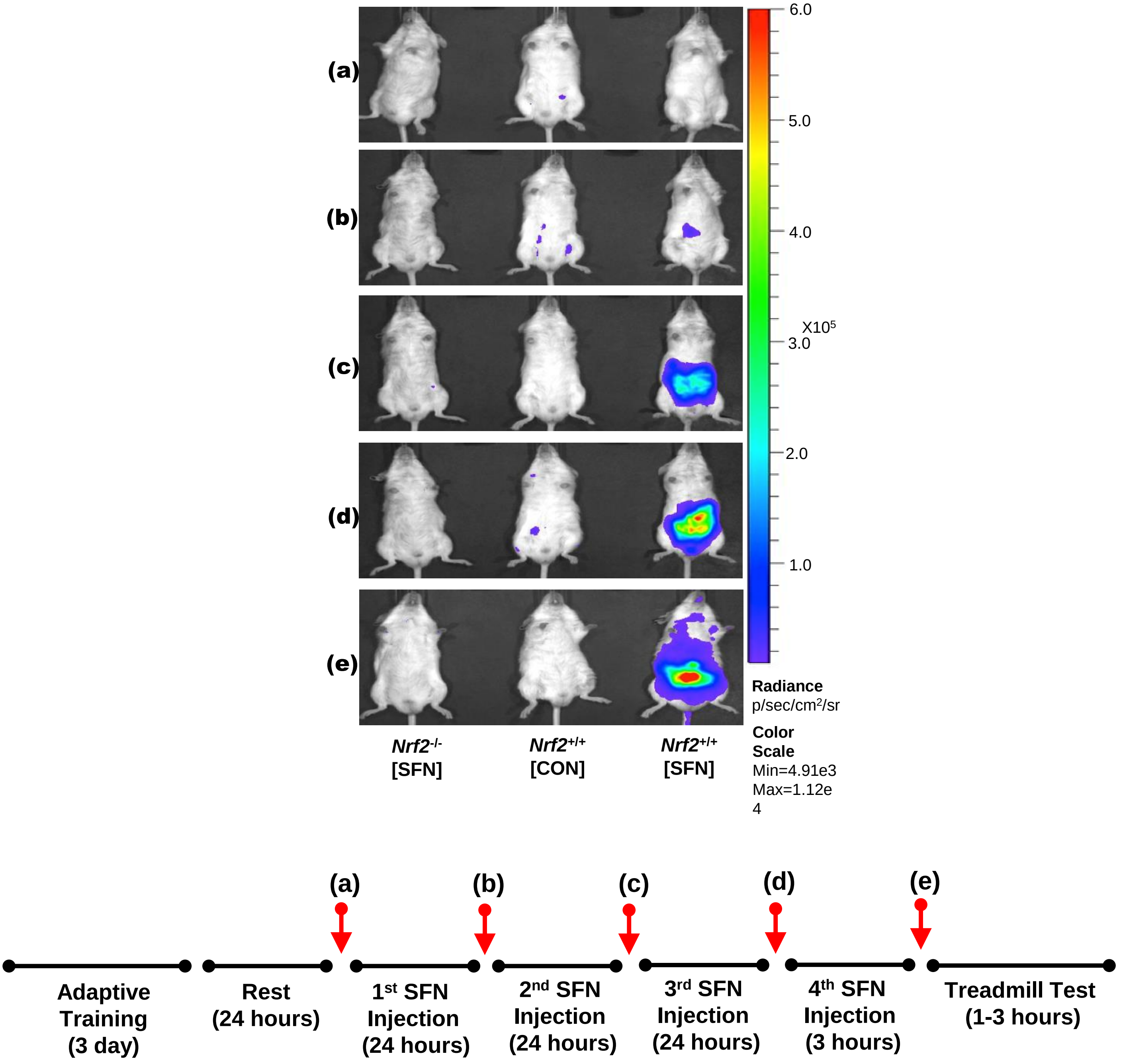

Supplementary figure S1.

Fig. S1. SFN-induced Nrf2-Luc activity detected using an In Vivo Imaging System and the results of genotyping in Nrf2 and OKD48 transgenic mice in the supine position. Time course of the Luciferase assay is shown (a) ~ (e)

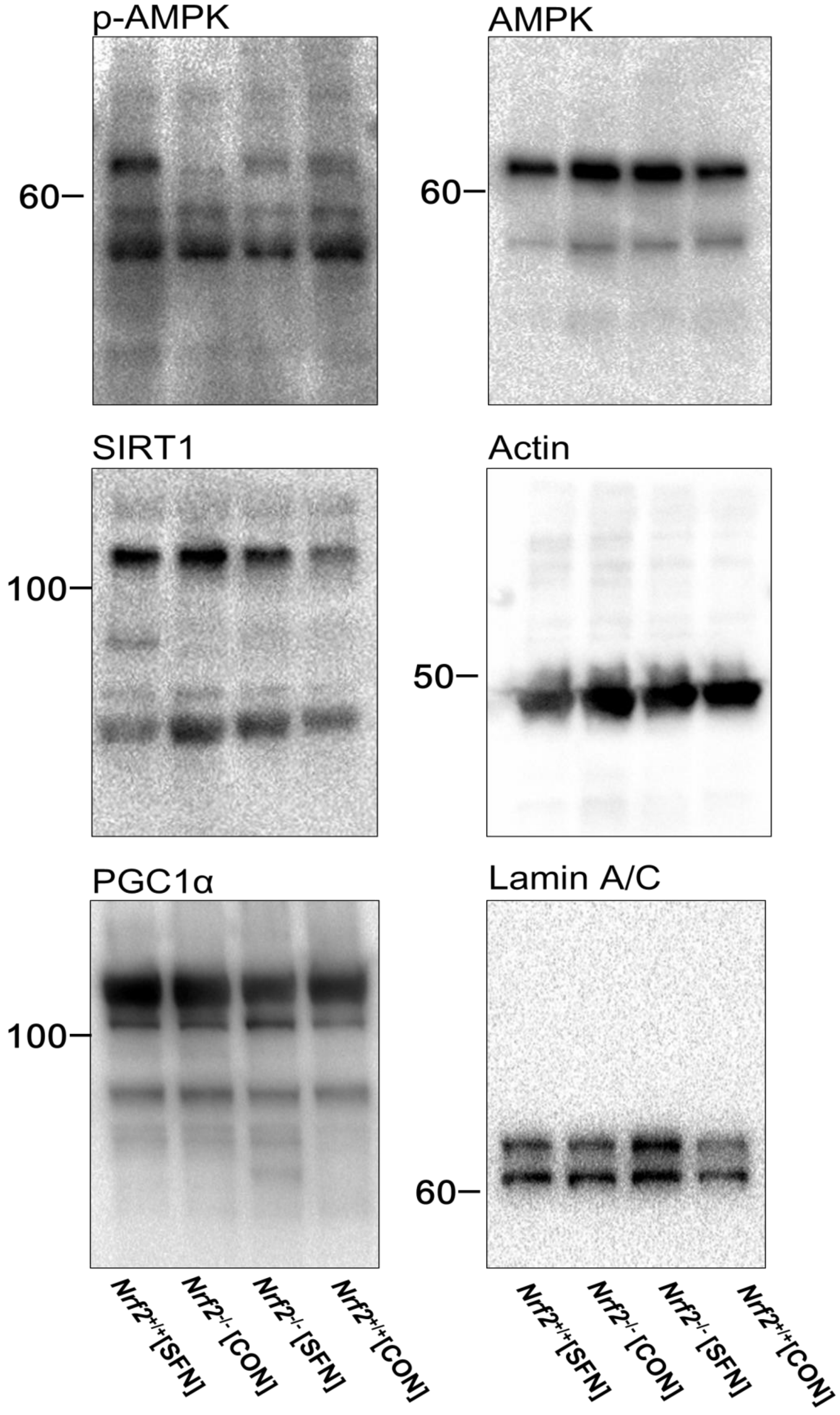

Supplementary figure S2. Full-length image of figure 3 (A)

The origin of these panels is identical with figure 3 (A) showing western blotting with phosphor-AMPK, AMPK, SIRT1, Actin, PGC1α, and Lamin A/C antibody
